# Complete Mitochondrial DNA Genome Variation in the Swedish Population

**DOI:** 10.3390/genes14111989

**Published:** 2023-10-25

**Authors:** Kimberly Sturk-Andreaggi, Martin Bodner, Joseph D. Ring, Adam Ameur, Ulf Gyllensten, Walther Parson, Charla Marshall, Marie Allen

**Affiliations:** 1 Department of Immunology Genetics and Pathology, Uppsala University, 751 08 Uppsala, Sweden; adam.ameur@igp.uu.se (A.A.); ulf.gyllensten@igp.uu.se (U.G.); 2 Armed Forces Medical Examiner System’s Armed Forces DNA Identification Laboratory (AFMES-AFDIL), Dover Air Force Base, DE 19902, USAcharla.k.marshall.civ@health.mil (C.M.); 3 SNA International, LLC, Alexandria, VI 22314, USA; 4 Institute of Legal Medicine, Medical University of Innsbruck, 6020 Innsbruck, Austria; martin.bodner@i-med.ac.at (M.B.); walther.parson@i-med.ac.at (W.P.); 5 Forensic Science Program, The Pennsylvania State University, University Park, State College, PA 16801, USA

**Keywords:** mitochondrial DNA, next generation sequencing, whole genome sequencing, nuclear mitochondrial DNA segment (NUMT), Sweden, population data

## Abstract

The development of complete mitochondrial genome (mitogenome) reference data for inclusion in publicly available population databases is currently underway, and the generation of more high-quality mitogenomes will only enhance the statistical power of this forensically useful locus. To characterize mitogenome variation in Sweden, the mitochondrial DNA (mtDNA) reads from the SweGen whole genome sequencing (WGS) dataset were analyzed. To overcome the interference from low-frequency nuclear mtDNA segments (NUMTs), a 10% variant frequency threshold was applied for the analysis. In total, 934 forensic-quality mitogenome haplotypes were characterized. Almost 45% of the SweGen haplotypes belonged to haplogroup H. Nearly all mitogenome haplotypes (99.1%) were assigned to European haplogroups, which was expected based on previous mtDNA studies of the Swedish population. There were signature northern Swedish and Finnish haplogroups observed in the dataset (e.g., U5b1, W1a), consistent with the nuclear DNA analyses of the SweGen data. The complete mitogenome analysis resulted in high haplotype diversity (0.9996) with a random match probability of 0.15%. Overall, the SweGen mitogenomes provide a large mtDNA reference dataset for the Swedish population and also contribute to the effort to estimate global mitogenome haplotype frequencies.

## 1. Introduction

Mitochondrial DNA (mtDNA) sequencing plays an important role in forensic casework, especially for the identification of human remains. Attributes of mtDNA such as a high copy number relative to nuclear DNA and matrilineal inheritance make the analysis a useful tool in decades-old cases that involve poor-quality DNA samples and no direct reference samples (e.g., [1,2,3,4,5]). Until recently, Sanger-type sequencing has been the “gold standard” for mtDNA analysis. However, this approach is expensive and labor-intensive, particularly for larger sample sets. For this reason, as well as for legal restrictions, forensic laboratories target the non-coding control region (CR) or the smaller hypervariable segments (HVSs) of the mtDNA rather than the entire mitochondrial genome (mitogenome). This results in limited discrimination power for mtDNA analysis due to common haplotypes observed in these smaller target regions. The most common European HVS (nps 16,024–16,365, 73–340) haplotype “263G 315.1C” is observed in approximately 1 in every 15 West Eurasian individuals in the European DNA Profiling Group (EDNAP) mtDNA Population (EMPOP) database (v4/Release 13) [6]. The use of the entire CR (nps 16,024–16,569, 1–576) slightly improves the discrimination power for mtDNA analyses, but full mitogenome data are required to completely resolve common haplotypes [7,8,9].

Mitogenome data can be efficiently and cost-effectively produced in forensic laboratories using next generation sequencing (NGS) techniques. Commercial NGS kits offer enrichment approaches that use small amplicons to target the mitogenome, which are amenable to a range of sample qualities (e.g., [10,11]), including reference-type samples and degraded DNA samples. Benchtop platforms such as the Verogen MiSeq FGx Forensic Genomics System (San Diego, CA, USA) and the Ion Torrent S5 System (Thermo Fisher Scientific, Waltham, MA, USA) allow practicable access to NGS technology. Although mtDNA analysis using NGS has been implemented in forensic laboratories [11,12,13,14], the usage of mitogenome data is also limited due to insufficient information on haplotype frequencies. At the time of publication, there are fewer than 5000 forensic-quality mitogenomes searchable in Release 13 of the EMPOP v4 database [6]. Without appropriate haplotype frequency information, the requisite match statistics that provide the evidentiary weight of the evidence in a forensic case cannot be accurately estimated [15].

To date, only two Swedish mtDNA population datasets are available for forensic use [16,17]. However, each high-quality dataset consists of fewer than 300 samples and only analyzed the mtDNA CR. Both of the studies that generated these datasets used Sanger-type sequencing for data generation with Lembring et al. targeting the ~600 bps of the two HVS regions [16], while Tillmar et al. analyzed the full CR [17]. These Swedish mtDNA datasets showed lower HVS/CR haplotype diversity compared to those from other populations in Europe. With the analysis of the entire mitogenome, the number of unique haplotypes could increase dramatically, potentially to a 100% resolution of common HVS/CR haplotypes, as observed in other West Eurasian populations [7,8,9,18]. Studies have, moreover, concluded that there is no significant substructure within Sweden based on mtDNA HVS/CR and Y-chromosomal markers [16,17,19,20], which has also been confirmed with autosomal DNA studies [21,22]. An exception to the observed homogeneity of the Swedish population is seen in the indigenous Saami located in northern Sweden [16,17,23]. Specifically, mtDNA haplogroup U5b1 is observed at a much higher frequency in Saami than in the larger Swedish population [24]. Individuals from northern Sweden, including the Saami, have been shown to be genetically similar to Finnish people [25]. Swedes from the rest of the country are more genetically similar to other neighboring European populations to the south and west, such as Germans, Danes, and Norwegians [26,27,28]. 

In this study, a further and more detailed evaluation of mtDNA variation in Sweden at the highest resolution based on high-quality mitogenome haplotypes from whole genome sequencing (WGS) data was performed [29]. WGS datasets are a largely untapped resource for mitogenomes. The use of these data allows for a rapid expansion of mitogenome reference databases with no additional laboratory processing costs, though there would be some cost associated with bioinformatic analyses and review of the data. Because the sequencing is untargeted, the greatest challenge to the mitogenome analysis of WGS data is nuclear mtDNA segments (NUMTs) [30]. Since reads from both mtDNA and NUMTs co-align to the mtDNA reference genome due to their homology, it can be difficult to distinguish between authentic point heteroplasmies (PHPs) and low-level variants associated with NUMTs [31,32,33]. The feasibility of high-quality mitogenome haplotype generation from WGS data was previously assessed by Sturk-Andreaggi et al. [29]. In this previous study, the authors demonstrated that reliable mitogenome haplotypes could be generated from WGS data using a 10% minimum variant frequency (VF) threshold [29]. Applying this frequency threshold, NUMT interference was negligible as the proportion of heteroplasmic haplotypes, and the maximum number of PHPs observed per individual, were consistent with previous high-quality mitogenome datasets [7,18,34]. Based on these results, the SweGen mitogenomes produced from WGS data with a 10% frequency threshold are appropriate for population-level investigations and haplotype frequency estimations for forensic purposes. The more than 900 Swedish haplotypes described in this study will substantially increase the number of high-quality mitogenomes available for forensic use, greatly improving the significance of mtDNA match statistics, which are dependent on the database size [15,35,36].

## 2. Materials and Methods

### 2.1. Samples

The SweGen WGS data [25] for the 942 Swedish individuals from the TwinGene project [37] were analyzed in order to generate mitogenome haplotypes. These unrelated individuals were initially selected for the SweGen project as a dataset representative of the population density distribution across Sweden. However, no regional information within Sweden for these samples was available for this study.

### 2.2. Haplotype Generation

WGS data were previously generated from the SweGen individuals as described in [25]. The WGS reads that aligned to the revised Cambridge Reference Sequence (rCRS) [38,39] were previously analyzed through a robust analysis pipeline to overcome NUMT interference [29]. In short, analysis was performed in CLC Genomics Workbench v12.0.1 with AQME v2.1.1 tools [40], requiring a minimum read depth of 100X and a 10% minimum VF threshold for variant calling. Haplotypes were subjected to independent reviews by at least two analysts followed by stringent quality control (QC) procedures as described in Taylor et al. [18] and Sturk-Andreaggi et al. [29]. To assist in the QC assessment of the mitogenome haplotype, the AQME Mitochondrial Haplogrouper tool predicted the mtDNA haplogroup based on Phylotree Build 17 [41,42].

In the present study, the SweGen samples with five or more positions below 100X (incomplete) and possible mixtures excluded from the analysis in the Sturk-Andreaggi et al. study were re-evaluated [29]. A 20X minimum read depth was applied along with the 10% frequency threshold for these previously incomplete samples when requiring 100X coverage of the mitogenome. NUMT interference was suspected for at least five of the seven of the samples classified as possible mixtures in [29] due to their low average read depths (<700X) and reduced proportions of mtDNA in relation to nuclear DNA (<0.012%). Therefore, the data for these seven samples with full 100X mitogenome coverage were also reanalyzed with the 10% frequency threshold. If any previously excluded mitogenomes were considered high-quality after re-evaluation, they were reviewed by two analysts and ultimately added to the 917 samples that were included in the initial SweGen mtDNA dataset [29].

To ensure the SweGen data represented a “random” sampling of the Swedish population, shared haplotypes (ignoring indels and heteroplasmy) were identified, and haplotype groupings were evaluated for relatedness [43]. Kinship coefficients were calculated based on the nuclear data from Ameur et al. [25] to determine if any samples with shared haplotypes were related as parent-offspring, siblings, or second-degree relatives (e.g., avuncular, grandparent/grandchild). There were two first-degree relatives with the same mitogenome haplotype identified in Sturk-Andreaggi et al. [29], and one sample of this maternally related pair was previously removed from the SweGen data included in this study. This assessment was repeated after reanalysis of the incomplete and possibly mixed samples to ensure no additional related pairs were present. If any additional related groups were found, only one mitogenome haplotype from related individuals was included in the final SweGen dataset. This additional QC measure was implemented to avoid potential bias introduced by the inclusion of close (maternal) relatives in an mtDNA population sample [43].

The final dataset was then submitted to EMPOP for additional QC checks and confirmation of the AQME haplogroup predictions [6,44].

### 2.3. Data Analysis

Outputs from the CLC Genomics Workbench were exported to Excel (Microsoft, Redmond, WA, USA), and analysis metrics were calculated, including average VF and average read depth. The metrics and other details were stored in Access (Microsoft), which was used to determine summary metrics. The distribution of PHPs, NUMT variants, and average read depth across the mitogenome was visualized using the circlize package v0.4.10 in R version 4.0.2 software [45,46]. Finalized haplotypes were uploaded to the Laboratory Information Systems Applications (LISA; Future Technologies Inc., Fairfax, VA, USA) database. Forensic and population genetic statistics, such as random match probability (RMP) and power of discrimination (haplotype diversity), were calculated based on pairwise comparisons performed in LISA. The comparisons, which ignored all indels, were performed using two approaches for matching: literal (e.g., a Y only matches a Y at a position) and pattern (e.g., a Y matches a C or T at a position). Additionally, summary statistics were calculated based on the two HVS regions (HVS1 nps 16,024–16,365 and HVS2 nps 73–340), CR (16,024–16,569, 1–576), and the entire mitogenome (1–16,569).

The SweGen dataset was compared to the two previous Swedish population datasets [16,17] at overlapping ranges (HVS for Lembring et al. [16] and CR for Tillmar et al. [17]). To ensure that the comparison of haplogroup composition was consistent (i.e., not impacted by the targeted region), haplogroups were assigned for HVS and CR range haplotypes using EMPOP v4 [44,47] for all datasets as applicable. Chi-squared tests with Yates’s correction were used to assess whether the haplogroup distributions for the three datasets were statistically different. An alluvial diagram was generated using RAWGraphs [48] to visualize the impact of the target region on haplogroup assignments for the SweGen haplotypes.

## 3. Results

### 3.1. Overall Performance

The mtDNA reads extracted from WGS data of 942 Swedish individuals were analyzed to produce mitogenome haplotypes for population genetics and forensic purposes. A total of 917 haplotypes were previously identified as being of high-quality [29]. Of these, 858 had complete 100X coverage across the mitogenome, while the remaining 59 were nearly complete, with less than five positions below the 100X threshold. A lower read depth threshold (20X) was applied to 17 previously incomplete haplotypes (five or more positions with less than 100 reads). As a result of this reanalysis, the full mitogenomes of 16 samples were covered with at least 20 reads. One incomplete haplotype was still observed with eight positions below the 20X read depth threshold. This sample was previously discussed in Sturk-Andreaggi et al. [29], as the five J1c2 haplogroup variants preceding the HVS2 C-stretch (nps 185–295) appeared to impact the coverage in this region. Nevertheless, two other incomplete haplotypes with the same haplogroup produced full mitogenome coverage with the 20X threshold, thus ensuring representation of this haplogroup in the dataset. The seven samples flagged as possible mixtures in [29] were also re-evaluated using the 10% threshold frequency. As a result, one haplotype, which had complete 100X coverage, was reclassified as a single-source profile since no mixed positions exceeded the 10% frequency threshold, except for one high-frequency (~50%) PHP, and all other metrics (e.g., average VF) were consistent with those of other single-source samples. The classification for the other six possible mixtures did not change, as these haplotypes could not be confidently classified as single-source. Additionally, the sample identified in [29] as a first degree relative of another sample in the dataset with a shared haplotype remained excluded from the dataset. No other maternally related individuals were identified in the SweGen samples included in the final mtDNA dataset. After reassessment, 934 forensic-quality Swedish mitogenomes were characterized (Table 1).

Mitogenome coverage at 100X was influenced by the number of mapped mtDNA reads, which had a linear relationship (R^2^ = 0.998) to the average read depth (Figure S1a). Overall, almost 280,000 reads on average were used to generate a mtDNA haplotype, ranging from approximately 25,000 to over 1.2 million reads. Complete 100X coverage of the mitogenome was obtained from samples with average read depths as low as 502X; however, the majority of the complete haplotypes had average read depths greater than 1275X (Figure S1b). With the exception of seven outliers, all nearly complete and incomplete haplotypes had average read depths of less than 1200X. In the study by Sturk-Andreaggi et al. [29], the average read depth was correlated to the proportion of mtDNA reads in relation to nuclear DNA reads in the WGS data rather than total WGS reads. Therefore, the nearly complete and incomplete samples had a lower mtDNA proportion in the WGS data (i.e., relative mtDNA copy number [49,50,51]) than the samples with complete 100X haplotypes.

**Figure 1:**
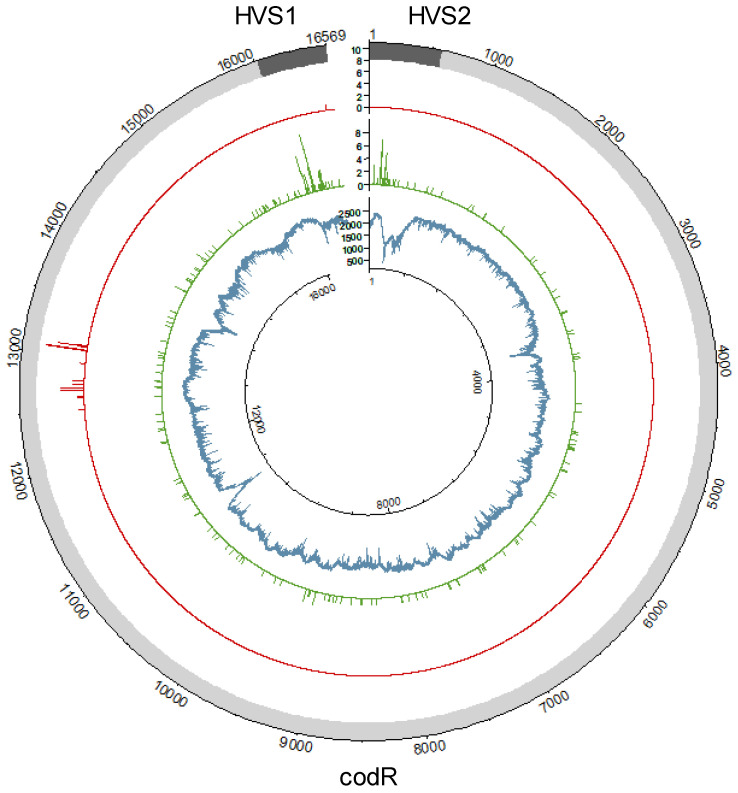
Distribution of the average read depth observed in a subset of 100 haplotypes (inner plot; blue), point heteroplasmies observed with the 10% frequency threshold (middle plot; green), and variants associated with nuclear mitochondrial DNA segments detected at 10% or higher frequencies (outer plot; red).

The distribution of coverage was evaluated for 100 samples, which were representative of the overall SweGen dataset (Table 1). The average read depth at each position was calculated based on the read depths observed in the data subset. This overall average read depth was 2328X and ranged from 411X to 2805X (Figure 1). Read depths were relatively consistent across the mitogenome, with approximately 10% variation in the overall average read depth and an interquartile range of 240X (Figure S2). However, there were 855 positions (5.2% of the mitogenome) with substantially lower read depths (less than 1883X). Most (760; 88.8%) of these low-coverage positions were localized to four regions: nps 217–598 (373), 3490–3634 (65), 10,891–11,043 (120), and 13,674–14,071 (202). The large proportion (43.6%) of the positions below 100X in the nearly complete and incomplete haplotypes occurred across nps 217–598 due to the presence of the three C-stretches (nps 303–309, 456–469, 568–576). In fact, several C-stretches are present in all four low-coverage regions, and HiSeq chemistry has previously been shown to exhibit poor sequencing in homopolymer regions [52]. As a result, reads in these regions were of generally poor quality, and thus read depths were reduced. Sequencing errors specific to polycytosine residues combined with the high-stringency mapping parameters employed to eliminate NUMTs are likely to have contributed to the low coverage observed in these regions.

The average frequency of the major nucleotide at all variant positions (average VF) was used to assess the quality of the haplotypes. This value is expected to exceed 98% in single-source, high-quality mitogenomes when PHP and LHP positions are ignored based on the level of observed background noise in the SweGen data. The average VF (excluding PHP and LHP) averaged 99.5% across the 934 SweGen mitogenomes (Table 1). Moreover, less than 1% of the SweGen haplotypes had average VFs below 98% (two complete, three nearly complete, and four incomplete). As shown in Figure S3, slightly lower average VFs were observed in the nearly complete (99.0%) and incomplete (98.5%) haplotypes compared to complete haplotypes (99.6%). This likely relates to the reduced average read depths observed in the non-complete (nearly complete and incomplete) haplotypes and increased observation of NUMT interference. In fact, the 31 NUMT-associated variants detected above the 10% frequency threshold were exclusively observed in haplotypes without full 100X coverage of the mitogenome. There were 13 NUMT variants in eight nearly complete haplotypes and an additional 27 NUMT variants detected in five incomplete haplotypes, all with average read depths of less than 550X. NUMT variants were detected above the 10% frequency threshold at multiple positions between nps 12,501 and 13,105 as well as at np 16,496 (Figure 1), which is consistent with the NUMT hotspots identified in [29]. Although NUMT-associated variants were not observed above the 10% frequency threshold in most haplotypes, NUMT interference was detectable above background noise (2% frequency) in nearly 40% (373) of the 934 SweGen mitogenomes. The presence of NUMT reads in the mtDNA alignments reduces the frequency of the major nucleotide at variant positions, thereby reducing the average VF. Since NUMT interference was correlated with lower average read depths, it is not unexpected that slightly lower average VFs were observed in the low-coverage data of the nearly complete and incomplete haplotypes (Table 1). 

### 3.2. Variants and Heteroplasmy

Overall, 23,857 variants including substitutions and indels, both homo- and heteroplasmic, were observed in the 934 SweGen mitogenome haplotypes, averaging 25.5 variants per haplotype. There was one sample with no differences from rCRS, belonging to haplogroup H2a2a1, and a maximum of 55 variants (i.e., differences from the rCRS) was detected in a T2f1a1 haplotype, which included nine deletions at nps 8281–8289. A total of 21,442 substitutions were observed at 1499 positions across the mitogenome. There were 355 deletions and 2060 insertions reported at 25 positions. 

There were 236 PHPs observed in 205 (21.9%) of the 934 SweGen mitogenome haplotypes. Most (177; 86.3%) of these haplotypes contained a single PHP. Two PHPs were observed in 25 haplotypes, and 3 haplotypes had three PHPs, which was the maximum number of PHPs observed in a single haplotype. The 236 PHPs were detected at 189 nucleotide positions (Figure 1). The majority (146; 61.9%) of the PHPs were detected in the coding region (codR), and these PHPs were located at 142 different positions. Of the 146 codR PHPs, 94.5% (*n* = 138) were observed once, and 4 were seen twice. In contrast, there were 90 CR PHPs detected at just 47 different positions. There were 13 CR positions at which PHPs were observed in more than one haplotype, ranging from two to nine occurrences. The most frequently observed PHP was 16192Y (*n* = 9), followed by 152Y (*n* = 7) and 16093Y (*n* = 6). Other heteroplasmic hotpots were observed at nps 146, 204, and 16,189 (all occurring five times). Overall, 181 (76.7%) of the PHPs involved transitions with 80 G/A (R) and 101 C/T (Y). There were eight heteroplasmic transversions: 593K, 955M, 4385W, 5625W, 13718S, 14020K, 16294S, and 16524W. There was one PHP (13105R with the G detected at 15.3%) reported in one haplotype, even though 13105G was also identified as a NUMT variant and was consequently removed from two incomplete haplotypes (with the G observed at 19.0% and 12.8%). As discussed above, these incomplete haplotypes had substantially lower average read depths (201X and 305X), resulting in increased NUMT interference, even above the 10% frequency threshold. The NUMT-associated 13105A was observed in-phase with other NUMT variants in this hotspot region [29]. Conversely, the haplotype that included the 13105R PHP had a high average read depth (>3000X) and no indication of NUMT interference, even below the 10% frequency threshold. 

Length heteroplasmy (LHP) was observed in 682 (73%) of the 934 SweGen mitogenome haplotypes. Although most of the haplotypes with LHP (*n* = 523) showed length variation in only one region, a portion of haplotypes had two (*n* = 150) or three (*n* = 9) regions exhibiting LHP. The region in which LHP was most often detected was the HVS2 C-stretch (*n* = 599), followed by the HVS1 C-stretch (*n* = 172), HVS3 C-stretch (*n* = 46), and AC-stretch at nps 513–524 (*n* = 17). Thirteen haplotypes displayed LHP in the C-stretches preceding nps 460 (*n* = 4), 960 (*n* = 5), 5899 (*n* = 2), and 8276 (*n* = 2). These LHP regions are well documented and typically ignored for forensic comparisons [6,15,44,53]. The LHP in the remaining three haplotypes was in other homopolymeric regions at which LHP is less frequently observed (i.e., the C-stretches preceding nps 356, 498, and 7471). However, the length variation observed in these regions was only evident in two haplotypes (i.e., the inclusion of a 356.1C and 498.1C) due to the reporting of the major length molecule. It is important to note that the reporting of PHPs in and around homopolymer regions was difficult due to post-homopolymer errors observed in HiSeq X sequencing [52]. As a result, low-level variants were consistently observed in the polyadenine stretches preceding the C-stretches of the HVS1 and HVS2 regions. In fact, 302M was observed in nearly all haplotypes, as was 16183M in samples with the 16189C variant, regardless of the complexity of the LHP. As a result, no haplotypes included a 302M or 16183M (or 16182M, etc.), and the major nucleotide at the position was reported. However, other PHPs in other flanking homopolymer regions were included in the haplotype when no or minimal length variation was observed. SweGen mitogenome haplotypes included 17 PHPs in or flanking the HVS1 C-stretch (at nps 16,188, 16,189, 16,192, 16,193, and 16,195). Additionally, PHPs at np 316 at the end of the HVS2 C-stretch and at np 955 at the beginning of another C-stretch at nps 956–960 were reported. 

### 3.3. Population Composition

A total of 821 mitogenome haplotypes were observed in the final SweGen mtDNA dataset (934 samples) when PHPs were treated literally and indels were ignored (Table 2). The most common mitogenome haplotype was observed seven times in the dataset (0.7% of the population), regardless of the match approach (i.e., literal or pattern) for comparisons (Table S1). This haplotype was assigned to haplogroup H2a1n (146C 263G 309.xC 315.1C 750G 951A 4659A 8860G 15326G 16354T). The next most common haplotype, which was seen six times (0.6%) in the dataset, was assigned to haplogroup U8a1a1a (73G 263G 282C 309.1C 315.1C 750G 1438G 1811G 2706G 3738T 4129G 4769G 5240G 6392C 6455T 7028T 7055G 8860G 9365T 9698C 10733T 11150A 11467G 11719A 12135A 12308G 12372A 13145A 14766T 15326G 16209C 16342C). A haplotype observed five times (0.5%) was assigned to another U haplogroup (U5b1b1a) and comprised the following variants: 73G 150T 263G 309.xC 315.1C 750G 1438G 2706G 3197C 4769G 5656G 7028T 7385G 7768G 8860G 9477A 10927C 11467G 11719A 12308G 12372A 12618A 13617C 14182C 14766T 15326G 16144C 16189C 16270T. There were five haplotypes seen four times in the SweGen dataset (0.43% of the population), including three haplotypes assigned to H haplogroups (H1c1a, H2a5, H6c) plus two haplotypes belonging to haplogroups V and W1a (Table S1). The remaining 67 other shared haplotypes (literal match approach and ignoring indels) were seen only two or three times in the SweGen population (Table S1). The observed haplotype frequencies correspond to a haplotype diversity of 0.9996 with the literal approach and 0.9993 when the pattern match approach is applied (Table 2). 

**Figure 2:**
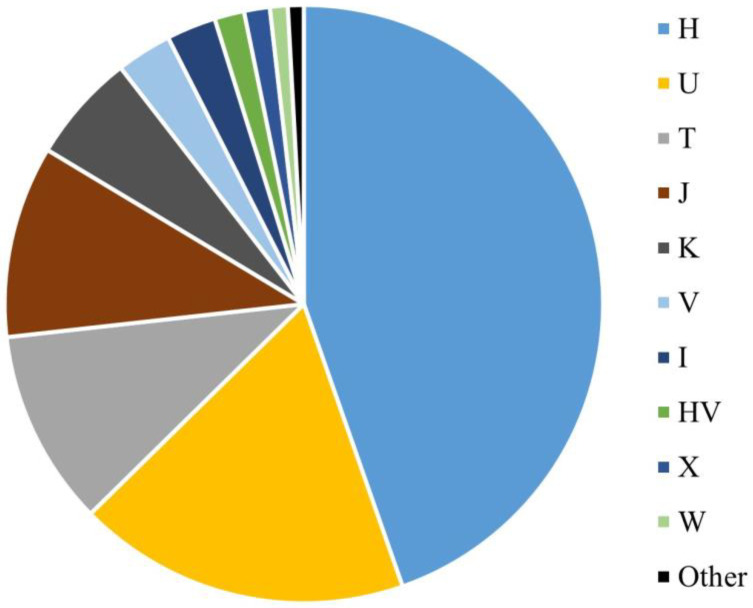
Macrohaplogroup breakdown for the SweGen mitochondrial genome data. The “Other” category (black) includes haplogroups D, G, M, N, and Z.

The mitogenome haplogroup composition of the SweGen dataset is similar to that of other Western European populations (Figure 2). Most haplotypes were assigned to haplogroup H (44.6%), which included subhaplogroups H1 (1.9%), H2a1n (1.1%), and H1b (1.0%). Haplogroup U was also observed in a large proportion of the SweGen samples (18.0%), with 12 (1.3%) haplotypes assigned to both U5b1b1a and U8a1a1a subhaplogroups (Table S1). In the SweGen dataset, U5b1 subhaplogroups were assigned to 3.9% (*n* = 36) of the haplotypes. Haplogroups T (10.6%) and J (10.4%) were also observed at high frequencies in the population, specifically subhaplogroups T1a1 (1.4%), T2b (1.2%), J1b1a1 (1.2%), and J2a1a1 (1.1%). Other common European haplogroups K (5.9%), V (3.0%), I (2.7%), HV (1.6%), X (1.4%), and W (1.0%) were represented (Table S1). There were a few haplotypes assigned to rarer European haplogroups N1a1a1a2 (0.2%) and Z1a1a (0.3%). In addition, several non-European haplogroups were observed once in the dataset (0.1%), including D4i, G3a3, and M1a1b1. These specific D and G haplogroups are common in Eastern Asia [54], while M1a1 haplogroups are predominantly observed in North and East Africa [55].

**Figure 3:**
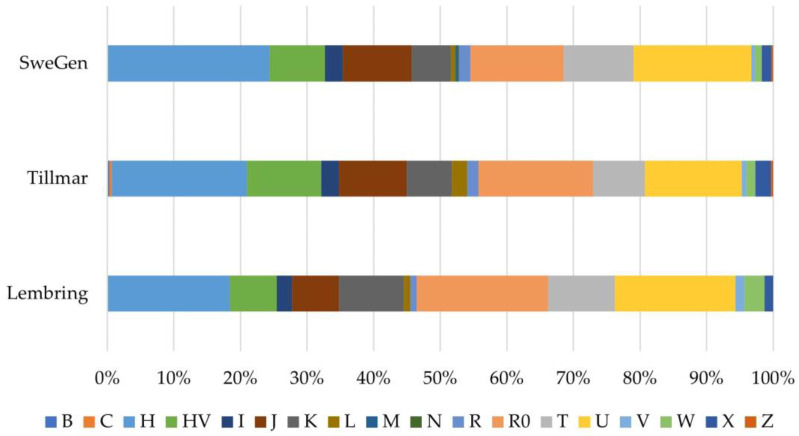
Macrohaplogroup breakdown of the SweGen, Tillmar et al. [17], and Lembring et al. [16] Swedish datasets based on the hypervariable segment regions (nps 16,024–16,365, 73–340), the largest overlapping range for all three datasets, using EMPOP v4 for haplogroup assignment.

The haplogroups observed in the SweGen dataset were consistent with previously published Swedish mtDNA data [16,17]. However, differences in the sequencing range of each Swedish dataset impacted the level of haplogroup refinement. For example, there was a high proportion of R0 haplogroups (18.4%) observed in the HVS data of Lembring et al. [16], whereas a greater proportion of H haplotypes (44.6%) were observed in the SweGen mitogenome dataset (Table S1). This is explained by the increased haplogroup resolution possible with sequencing larger portions of the mitogenome, resulting in the refinement of the R0 haplotypes from HVS data to H haplogroups with entire mitogenomes (Figure S4). A comparison of updated haplogroup predictions based on HVS regions confirmed this since the updated haplogroup predictions utilized the same prediction tool and target region for the three Swedish datasets (Figure 3). This analysis showed no significant difference in HVS haplogroup frequencies between the three Swedish population datasets (*p* > 0.13), although the SweGen still had a slightly higher proportion of H haplogroups than R0, in contrast to the other two Swedish datasets. When comparing the CR haplogroup frequencies between the Tillmar et al. [17] and SweGen datasets, a range that was not sequenced for the Lembring et al. dataset [16], there was no significant difference (*p* = 0.88; Figure S5). Minor differences in haplogroup proportions are not unexpected and likely the result of sampling variation as well as the size of the populations, as the SweGen dataset is over three times larger than the other two Swedish datasets. 

## 4. Discussion

The observed heteroplasmy in this dataset was consistent with the findings in previous studies that produced forensic-quality mitogenome data [7,18], providing the basis for applying the 10% minimum VF threshold, as discussed in Sturk-Andreaggi et al. [29]. Furthermore, the SweGen mitogenomes presented here are notably different than those from questionable datasets that contain high numbers of PHPs per haplotype, codR heteroplasmic hotspots, and high frequencies of heteroplasmic transversions [56]. There were six heteroplasmic hotpots (observed five times or more), all of which were observed in the CR and were previously reported by Irwin et al. [57]. Of note, 16192Y was observed more frequently in this dataset compared to other studies. Due to its presence in a homopolymer region, heteroplasmy detected at np 16,192 was scrutinized to ensure that reported PHPs were not caused by misalignment. It is therefore likely that the higher PHP rate at np 16,192 seen in the SweGen dataset is the result of a high frequency of U5 haplogroups (10.3%) in which 16192T is a diagnostic mutation [42]. In terms of LHP, it is important to understand the impact of the sample preparation method and sequencing platform on the homopolymeric regions when analyzing NGS data [58]. These factors may impact the interpretation and reporting of these regions, such as the inclusion of PHPs or reporting of the major length molecule [53]. Procedures then need to be adjusted based on the methods used for the data generation to ensure the production of high-quality haplotypes.

The mitogenome haplotype diversity observed In the SweGen dataset (0.9996) is similar to that of other Western European populations [59,60]. Compared with the U.S. populations of European ancestry [7,18], the Swedes exhibit a higher proportion of shared haplotypes. As expected, the number of unique haplotypes decreases when considering smaller ranges (from 629 to 372 for the CR and 324 for the HVS), resulting in lower RMPs and haplotype diversities than for the entire mitogenome. The CR haplotype diversity for the SweGen population (0.9963 literal, 0.9943 pattern) was consistent with that of the Tillmar et al. Swedish CR dataset (0.996) [17]. In addition, the HVS population statistics for the SweGen data using the pattern approach (RMP of 1.14% and haplotype diversity of 0.9897) were similar to the findings of Lembring et al. (RMP of 1.39% and haplotype diversity of 0.9895) [16]. Regardless of range, when the pattern match approach was used for comparisons, the RMPs increased, while the haplotype diversities decreased slightly due to the increase in non-unique haplotypes.

Ameur et al. noted a small representation of Finnish ancestry in the nuclear DNA of the SweGen individuals [25], which was further supported by the mtDNA analyses. Haplogroups U5b1 and W1a, which are common within Finland (both ~6%) [61], were observed in 3.9% and 1.7% in the SweGen dataset, respectively. Additionally, haplogroup U5b1 was observed in higher proportions in northern Swedes [17], specifically in the Saami population [24]. Interestingly, U5b1 has also been documented in Viking Age Scandinavians [62]. We expected to observe this haplogroup in the SweGen dataset as it was sampled to represent the genetic diversity within the Swedish population, though no regional information was known for these samples. Ameur et al. also detected an East Asian bias observed in the SweGen nuclear data [25], which was also seen in the mitogenome data with two East Asian mitogenome haplotypes observed (i.e., D4i and G3a3 haplotypes). The presence of non-European maternal lineages in the SweGen dataset is not unexpected given the findings from previous studies [17,23]. In particular, Lappalainen et al. showed slightly elevated frequencies of East Asian and Native American haplogroups as well as African and Near East influence [23]. The observation of non-European haplogroups in the Swedish population is likely the result of increased immigration from more distant countries during the end of last century [63]. 

## 5. Conclusions

The SweGen WGS dataset was used for the determination of mtDNA haplotype frequency estimates. The high haplotype diversity and haplogroup distribution observed in the SweGen population were consistent with existing Swedish datasets [16,17]. Moreover, the observation of haplogroups characteristic of northern Swedish and Finnish populations in the SweGen data confirmed this finding from the analysis of the nuclear DNA [25]. The nuclear DNA from the SweGen WGS data was also beneficial in this study as it was readily available for kinship analyses to identify close maternal relatives with shared mitogenome haplotypes [43]. Although it is possible to identify distant relatives from WGS data [64], it may be necessary to determine the degrees of relatedness that should be included in mtDNA reference datasets to reflect the haplotype distribution in the population properly. In the end, over 900 high-quality mitogenome haplotypes were generated from WGS data with no additional costs, except for those associated with the analysis. This large dataset of Swedish mitogenomes is now available for population genetic studies as well as forensic applications. 

## Figures and Tables

**Table 1:** Summary of analysis metrics for the final SweGen mitochondrial DNA (mtDNA) dataset. Haplotypes are separated based on mitochondrial genome (mitogenome) coverage at 100X with complete, nearly complete (four or less positions below 100X), and incomplete (more than four positions below 100X) classifications. The average (avg) major nucleotide frequency is determined for all variant positions (All), as well as excluding both length and point heteroplasmy (No HP). The “Complete” 100X coverage group includes the one haplotype classified as a possible mixture in [29]. The data subset is a group of 100 SweGen samples that were selected as a smaller representation of the overall SweGen data. The subset was used to evaluate the distribution of coverage at each position in the mitogenome.

100X Coverage	Sample Count	Avg Mapped mtDNA Reads	Avg Read Depth	Avg Major Nucleotide Frequency	
				All	No HP
Complete	859	297,369.6	2363.8	98.0	99.6
Nearly Complete	59	82,393.4	652.5	97.5	99.0
Incomplete	16	69,264.9	538.4	95.7	98.4
***All***	***934***	***279,882.2***	***2224.4***	***98.0***	***99.5***
*Subset*	*100*	*288,141.7*	*2271.6*	*97.9*	*99.5*

**Table 2:** Haplotype diversity estimates for the SweGen population (*n* = 934). The number of haplotypes was determined based on pairwise comparison performed with both literal and pattern matching for point heteroplasmies. The summary statistics including random match probabilities (RMP) were calculated for three different ranges: hypervariable segments (HVS; nps 16,024–16,365, 73–340), control region (CR; nps 16,024–16,569, 1–576), and the entire mitochondrial genome (mtG; nps 1–16,569). Indels were ignored in all comparisons.

Range	Match Type	Total Haplotypes	Unique Haplotypes (Proportion Unique)	Observed RMP (%)	Empirical RMP (%)	Haplotype Diversity
HVS	Literal	531	393 (74.0%)	0.71	0.60	0.9940
	Pattern	473	324 (68.5%)	1.14	1.03	0.9897
CR	Literal	583	447 (76.7%)	0.47	0.37	0.9963
	Pattern	524	372 (71.0%)	0.67	0.57	0.9943
Mitogenome	Literal	821	746 (90.9%)	0.15	0.04	0.9996
	Pattern	750	629 (83.7%)	0.17	0.07	0.9993

## Data Availability

The SweGen mitogenome haplotypes are presented in Table S1 and are available upon request.

## Supplementary Materials

The following supporting information can be downloaded at: https://www.mdpi.com/article/10.3390/genes14111989/s1, Table S1: The haplogroup breakdown using the literal match approach for the 934 SweGen haplotypes included in the final mitochondrial genome dataset; Figure S1: Graphical description of the average read depths observed in the SweGen dataset; Figure S2: The distribution of read depth for the 16,569 positions of the mitochondrial genome based on the average observed in a subset of 100 representative SweGen haplotypes; Figure S3: The box-and-whisker plot presents the distribution of average variant frequency for each coverage classification group; Figure S4: Alluvial diagram representing the change in haplogroup assignments for the SweGen dataset for hypervariable segment (HVS) regions to control region (CR) and the CR to entire mitochondrial genome (mitogenome). Colored bands denote haplotypes that were assigned to different haplogroups between one region and the next region (i.e., HVS to CR, CR to mitogenome), whereas gray bands indicate no change in the haplogroup assignment.; Figure S5: Macrohaplogroup breakdown the SweGen and Tillmar et al. [17] Swedish datasets based on the control region (nps 16,024–16,365, 73–340) using EMPOP v4 for haplogroup assignment.
